# A method for non-invasive genotyping of APC^min/+ ^mice using fecal samples

**DOI:** 10.1186/1480-9222-14-1

**Published:** 2012-01-30

**Authors:** Erin L Symonds, Michael Fenech

**Affiliations:** 1 Nutrigenomics and Nutrigenetics, CSIRO- Food and Nutritional Sciences, Adelaide, South Australia; 2 Nerve-Gut Research Laboratory, Royal Adelaide Hospital, Level 1 IMVS Building, Frome Road, Adelaide, South Australia, 5000

**Keywords:** APC^min/+^, feces, genotyping, cancer, non-invasive

## Abstract

The APC^min/+ ^mouse is commonly used in cancer research and is just one of many genetically altered models that is currently being developed. With high numbers of breeding programs, it is important to have a simple method that can be used to genotype the mice non-invasively. Here we report a reproducible method for genotyping mice with DNA extracted from fecal samples. Comparison of fecal results with those obtained from intestinal tissue DNA and clinical outcome (presence/absence of tumors) showed this technique to have 100% accuracy. This non-invasive method of genotyping may be applied to other transgenic mouse models.

## Introduction

With advances in molecular genetics, there are large numbers of transgenic and knock-out mice being developed. The most common method for genotyping these mice is with DNA isolated from tail, ear or toe tissue, or from blood. These collection techniques however are invasive procedures and are not appropriate for repeat sampling. Recent publications have shown that DNA can be isolated from fecal samples, which allows for non-invasive genotyping [1,2].

In this work we describe a method for genotyping APC^min/+ ^and wild-type mice with DNA isolated from fecal pellets. The APC^min/+ ^mouse is a model for familial adenomatous polyposis, with the mice highly susceptible to spontaneous intestinal adenoma formation at an early age, and anaemia as a secondary condition [3]. This mouse model is therefore commonly used in cancer research. It has a chemically-induced dominant mutation (base substitution mutation) in the adenomatous polyposis coli (APC) gene which is considered a tumor suppressor gene [4]. It has been shown that the main mechanism for tumor induction is loss of the wild-type (Apc+) allele (ie. loss of heterozygosity) [5,6].

The aims of this study were to assess the sensitivity, specificity and reproducibility of genotyping APC^min/+ ^mice and their wild-type littermates with DNA isolated from their fecal samples.

## Results and discussion

This study has shown that APC^min/+ ^mice and their wild-type litter mates (> 3 wk) can be genotyped using DNA isolated from whole fecal samples without the need for isolation of the colonic cells from the rest of the fecal mass. The collection technique is non-invasive to the mouse and therefore repeatable. Fecal pellet collection from mice aged greater than 3 wk was very simple, with the majority of mice providing a sample within 1 min. Collection of feces from mice younger than the weaning age (3 wk) was difficult due to their milk diet, and resulted in a poor DNA yield as the sample was small. DNA extraction from a mouse (> 3 wk) fecal pellet resulted in a yield of 20 - 350 ng/μL (average 75 ± 4 ng/μL (mean ± standard error)), and a purity (determined with the nanodrop OD_260_:OD_280_) of 2.02 ± 0.01. Following successful amplification of the DNA, running the PCR products on the gel allowed the determination of the genotype of the mouse.

To determine the accuracy of the genotyping with fecal samples, 28 wild-type and 28 APC^min/+ ^mice were genotyped with both fecal and small intestinal tissue DNA samples. There was 100% agreement between the results (Table 1). Another validation of the genotyping results was completed by the presence or absence of small intestinal tumors in wild-type and APC^min/+ ^mice (n = 40/group). All mice established as APC^min/+ ^with the fecal DNA samples were found to have tumors in their small intestine at age 13 wk, compared to none of the wild-type mice (Table 1). This validation shows 100% sensitivity and specificity of the genotyping.

**Table 1:** Comparison of fecal genotyping result to other methods

	Genotype determined from fecal DNA	
Alternative technique	Wild-type	APC^min/+^
Presence of intestinal tumors	0/40	40/40
Intestinal tissue genotype positive for wild-type	28/28	0/28
Intestinal tissue genotype positive for APC^min/+^	0/28	28/28

The reproducibility of the genotyping with fecal samples was assessed by collecting and analyzing DNA from fecal pellets from mice on multiple occasions (with at least one week between collections). All samples gave the same genotype every time tested, showing the method to be reproducible (data not shown).

## Methods

APC^min/+ ^and wild-type (APC^+/+^) mice were produced in a breeding program that involved mating male APC^min/+ ^(Jackson Laboratories, Bar Harbor, ME) with female C57BL/6 mice (ARC, Perth, Australia). All methods were approved by the CSIRO animal ethics committee (Adelaide, South Australia).

### DNA isolation

Fresh fecal samples were collected from the offspring (aged 3 - 13 wk) by placing the mouse in an empty cage until a pellet was passed. One fecal pellet per mouse was adequate for analysis. The DNA was isolated while the sample was fresh using the Qiagen Blood and Tissue Kit (Qiagen, Victoria, Australia). Briefly, 180 - 380 μL tissue lysis buffer was added to the fecal sample (depending on the size) along with 20 μL proteinase-K. The sample was incubated at 55°C for 2.5 h (vortexed every 45 min), followed by the addition of a further 200 μL lysis buffer. This resulted in the majority of the fecal pellet being dissolved and filtering of the sample was not performed. The DNA was precipitated with ethanol and purified with the Qiagen spin column according to the manufacturer's instructions. DNA was eluted in 100 μL AE buffer and quantified with a nanodrop (NanoDrop Technologies, Wilmington, DE). A sub-group of mice aged 13 wk were culled and small intestinal tissue was collected and stored at -80°C. DNA was isolated from the tissue following the above protocol, but the tissue was first lysed in 180 μL tissue lysis buffer with 20 μL proteinase-K overnight at 36°C.

### Genotyping

**Figure 1:**
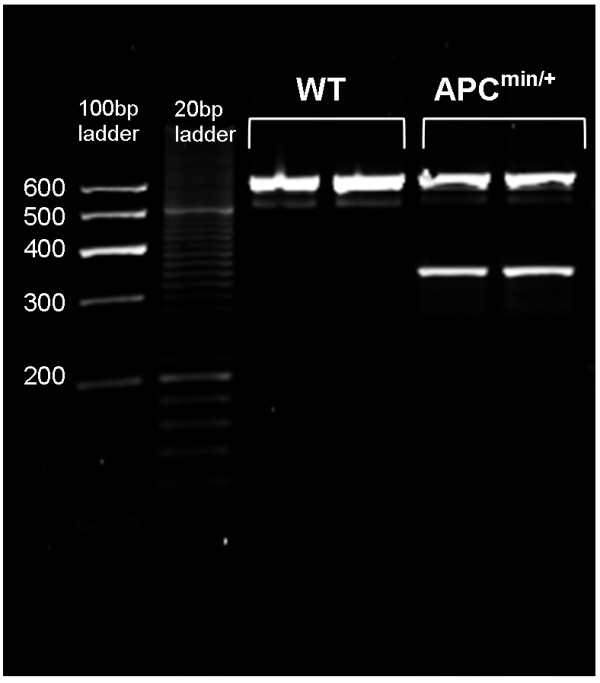
**Genotyping of APC^min/+ ^mice with fecal DNA**. Analysis of PCR products with agarose gel electrophoresis, showing products of the predicted sizes (Lane 1- 100 bp ladder; Lane 2- 20 bp ladder; Lane 3 and 4- PCR products from mice with the wild-type allele, 619 bp; Lane 5 and 6- PCR products from mice with the APC^min/+ ^allele, 619 bp and 331 bp).

For each DNA sample, real-time PCR was set up containing three primers (a specific primer for wild-type and APC^min ^and a common antisense primer; Table 2[7]) to detect each allele. Real time PCR was done in a final volume of 20 μL/sample, using 10 μL SYBR^® ^Green PCR Master Mix (Applied Biosystems, Victoria, Australia), 0.25 μL wild-type primer, 0.25 μL APC^min ^primer, 0.5 μL common antisense, and 40 ng DNA. A negative PCR control contained all reagents except DNA. Real time PCR was performed using the Applied Biosystems 7300 Real-Time PCR System, with the following cycling conditions: 10 min at 95°C, followed by 40 cycles of 95°C for 30 sec, 55°C for 30 sec, and 72°C for 1 min. The dissociation stage was performed by 95°C for 15 sec, 55°C for 30 sec, and 95°C for 15 sec. PCR products were run on 2% (w/v) agarose gel in 0.5 × TAE buffer (Tris, acetic acid, ethylenediaminetetraacetic acid) containing gel red (Jomar Diagnostics, Stepney, South Australia). Wild-type mice had a single band (619 bp), while APC^min/+ ^mice had two bands (331 bp and 619 bp, Figure 1)

**Table 2:** Polymerase chain reaction primers [7]

Primer	Sequence
Wild-type	5' GCC ATC CCT TCA CGT TAG 3'
APC^min^	5' TTC TGA GAA AGA CAG AAG TTA 3'
Common antisense	5' TTC CAC TTT GGC ATA AGG C 3'

### Analysis

Fecal genotyping results were assessed for sensitivity and specificity by comparing the results to those obtained from intestinal tissue DNA, and to the presence or absence of intestinal tumors in mice. The reproducibility of the fecal genotyping technique was assessed by collecting additional fecal pellets from mice, with at least one week between collections. Fecal samples were collected from wild-type (n = 21) and APC^min/+ ^mice (n = 29) on two to eleven occasions and analyzed for genotype.

## Conclusions

In conclusion, this technique is suitable for genotyping APC^min/+ ^mice and their wild-type littermates. The genotype results using fecal DNA were consistent with that found from DNA isolated from small intestinal tissue, and also consistent with the presence or absence of intestinal tumors. It is a simple and reproducible test that can be developed for other genetically altered mouse models. Its non-invasive nature will reduce physical injury and anxiety of the mouse which will improve animal welfare.

## Abbreviations

APC: adenomatous polyposis coli; CSIRO: Commonwealth Scientific and Industrial Research Organisation; PCR: polymerase chain reaction.

## Competing interests

The authors declare that they have no competing interests.

## Authors' contributions

ELS designed and conducted the trial, performed the data analysis and drafted the paper. MF was involved with data analysis and interpretation, and revised the draft paper. All authors read and approved the final manuscript.
